# Growth and developmental outcomes of infants with hypoxic ischemic encephalopathy

**DOI:** 10.1038/s41598-023-50187-0

**Published:** 2023-12-28

**Authors:** Joonsik Park, Sook Hyun Park, Chloe Kim, So Jin Yoon, Joo Hee Lim, Jung Ho Han, Jeong Eun Shin, Ho Seon Eun, Min Soo Park, Soon Min Lee

**Affiliations:** Department of Pediatrics, Yonsei University College of Medicine, 211 Eonjuro Gangnam-gu, Seoul, 06273 Republic of Korea

**Keywords:** Medical research, Paediatric research

## Abstract

Despite advances in obstetric care, hypoxic ischemic encephalopathy (HIE) remains a significant disease burden. We determined the national trends of HIE prevalence, therapeutic hypothermia (TH) use, mortality, and outcomes from 2012 to 2019. This study included term infants diagnosed with HIE between 2012 and 2019 from the National Health Insurance Service database. The prevalence of HIE was 2.4 per 1000 births without significant change during the period. TH was performed in approximately 6.7% of infants with HIE, and the annual variation ranged from 2.4 to 12.5%. The mortality among all term infants with HIE was 4.6%. The mortality rate among infants with HIE and TH significantly declined from 40 to 16.9% during the eight years. Infants with TH had higher mortality, increased use of inhaled nitric oxide, and more invasive ventilator use, indicating greater disease severity in the TH group. Infants with TH also showed significantly poorer outcomes, including delayed development, cerebral palsy, sensorineural hearing loss, and seizure, compared to infants without TH (p < 0.0001). With the increasing application of TH, mortality and developmental outcomes among infants with HIE have been improving in the past eight years in Korea. Further efforts to improve outcomes should be needed.

## Introduction

Hypoxic Ischemic Encephalopathy (HIE), caused by impaired cerebral blood flow and oxygen delivery to the brain, is a severe birth complication affecting term infants^1^. Despite advances in obstetric care, HIE continues to affect 1.5 infants per every 1000 births worldwide and accounts for a significant disease burden in the developed country^2^.

Since approved by the United States Food and Drug Administration in 2005, therapeutic hypothermia (TH) has been the standard treatment for neonates at a gestational age of 35 weeks or more with moderate to severe HIE within the first 6 h after birth^3^. In the US, 2.4 per 1000 births are diagnosed with HIE, and TH is applied to 10.9% of those with HIE^4^. In the United Kingdom, 1.2–3.0 per 1000 births are diagnosed with HIE, and TH is applied to 41–67%, with 0.98 deaths per 1000 noted^5,6^. In Sweden, 1.4 per 1000 births have HIE, and 47% have TH^7^. Clinical trials have shown that TH reduces the risk of death in infants with HIE compared to control groups (10% vs. 20–30%)^5,6,8^. In Korea, TH has been adopted as standard therapy since 2012 and is covered under national insurance^9^. A single center from Korea reported that 10% of infants die and 42% have abnormal neurological outcomes among the infants diagnosed with HIE who undergo TH^10^: population-based national data about HIE and TH have yet to be reported in Korea.

TH for newborns with HIE has been shown to improve neurodevelopmental outcomes significantly. Results from a Cochrane Review stated that hypothermia at 33–34 °C for 72 h reduced death or significant neurodevelopmental disability among survivors at 18 or 24 months of age. In particular, TH decreased the risk of neuromotor and developmental delay and that of cerebral palsy among the survivors^8^. TH has also been shown to have a neuroprotective effect in children aged 6–7 years^11^. Data from the Total Body Hypothermia for Neonatal Encephalopathy trial showed that more children in the cooled group than the control group survived with an intelligence quotient more significant than 85^12^. Furthermore, more children in the cooled group demonstrated regular neurological exams, and fewer had cerebral palsy. MRI results have also shown a lower incidence of brain injury in the cooled population than in the non-cooled population (36% vs 66%)^13^.

In 2023, it is now well-established that TH yields superior outcomes and should be employed whenever indicated. Despite receiving TH, up to 29% of neonates with HIE still develop adverse effects^14^. The results of HIE included high mortality or severe disabilities, such as mental retardation, epilepsy, and cerebral palsy, in 40% to 60% of such infants, attributed to the severity of the disease^15–17^. While TH has benefits, its safety and efficacy in diverse circumstances remain uncertain, particularly in newly introduced countries. In the HELIX trial of 2021, the authors emphasized the need for special attention until optimal care and effective cooling are available^18^.

We aimed to assess the trends in HIE prevalence, TH application, mortality, and associated outcomes over a sequential 8-year period, starting from the early introduction in 2012, using a population-based national database in Korea.

## Methods

### Patients and data source

This study analyzed the data of term infants diagnosed with HIE (International Classification of Diseases-10 code: G93.1) between 2012 and 2019 from the National Health Insurance Service (NHIS) database. For almost all Korean residents, healthcare claims data, such as diagnostic codes, diagnostic test costs, and administered procedures, are linked to the National Health Screening Program for Infants and Children database. Information on birth weight or gestational age was obtained by the ICD-10 codes input by the hospital or by the “Questionnaire” administered as part of the National Health Screening Program for infants and children. In Korea, the indications for TH are primarily based on the recommendations by the Korean Neonatal Society as a minimum birth weight range of 1800–2000 g, gestational age of 35 weeks, and moderate to severe Sarnat stage^9,19^. But, due to discrepancies between international guidelines, domestic recommendations, and hospital variations over a research period of 8 years, there was a potential bias depending on the choice of subjects in this study. In addition, in ICD-10 codes, cases with birth weight exceeding 1800 g are mixed in the P07.17 code (1750–1999 g), making it impossible to distinguish them separately. Therefore, this study exclusively focused on term infants and a birth weight exceeding 2000 g. We excluded preterm infants if the questionnaire confirmed preterm status or those with ICD-10 codes P07.2 and P07.3. We excluded birth weight below 2000 g for term infants with ICD-10 codes P07.0–07.17. We used birth certificate data from Statistics Korea to estimate the prevalence of HIE and TH incidence (https://kosis.kr/statisticsList).

The National Health Screening Program for infants and children in Korea was launched in 2007 to monitor current health issues. It has since been a successful part of the primary clinical service^20^. The program provides population surveillance that includes medical history, physical examination, anthropometric measurements, screening for visual acuity, developmental screening via the Korean Developmental Screening Test (K-DST), oral examination, and questionnaires on anticipatory guidance. The study population of infants had their first visits at 4–6 months of age, second visits at 9–12 months, third visits at 18–24 months, fourth visits at 30–36 months, fifth visits at 42–48 months, and sixth visits at 54–60 months, based on the chronological age, not the corrected age. The K-DST screening test verifies whether infants have achieved normal neurodevelopmental status in six domains (gross/fine motor, cognition, communication, social interaction, and self-control)^21,22^. Tests were administered according to the child’s corrected age during the clinic visits. Results were categorized into four groups based on standard deviation (SD) scores (a score below −2 SD = “further work-up”, between −2 and −1 SD = “close observation,” between −1 and 1 SD = “at a peer level”, and above 1 SD = “at a high-level”). For children in the close observation group, short-term checkups were recommended. Scores below − 1 SD were set as a critical cutoff to screen for developmental delays, indicating that these infants require further evaluation and follow-up tests. Additionally, there were some positive responses to questions that were considered red flags for clinically significant neurodevelopmental disorders, such as cerebral palsy, language delay, and autism spectrum disorders, and these infants were referred to medical specialists. The complications associated with HIE were identified using ICD-10 codes input by the hospital including delayed development (DD) (R62.9), cerebral palsy (CP) (G80), autism spectrum disorders (ASD) (F84.9), sensorineural hearing loss (SNHL) (H90.5), attention deficit hyperactivity disorder (ADHD) (F90.0), Blindness (H54.0) and Seizure disorder (G40 or R56.8). The Disability Rating Standards were established through an official announcement by the Ministry of Health and Welfare of the Republic of Korea, as per Notification No. 2013-56 (April 3, 2013) (http://www.mohw.go.kr/upload/viewer/skin/doc.html?fn=1365062659892_20130404170421.hwp&rs=/upload/viewer/result/202310/).

### Statistical analyses

Baseline infant characteristics were expressed as percentages for categorical variables. The cohort was stratified according to birth year. A test was used to compare the neonatal features and complications between the groups. Logistic regression models were used to determine the significant changes in the incidence of complications, and odds ratios (ORs) and 95% confidence intervals (CIs) were calculated for each risk factor associated with mortality and morbidity. The Cochran-Armitage trend test was used to test for linear trends. All analyses were performed using SAS v 9.4 (SAS Institute, Cary, North Carolina). A *p*-value < 0.05 was considered statistically significant.

### Ethics statement

This study used NHIS-NSC data (NHIS-2022-1-120) maintained by the NHIS. The authors declare no conflicts of interest with NHIS. In this study, all identifiable variables, including claim-, individual-, and organizational-level identification numbers, were randomly re-generated by the NHIS database to protect patient privacy. The study protocol was approved by the Institutional Review Board of Gangnam Severance Hospital, Yonsei University School of Medicine (No. 3-2021-0221). Informed consent was waived by Yonsei University School of Medicine owing to the retrospective study design. The relevant guidelines and regulations performed all methods.

## Results

**Figure 1:**
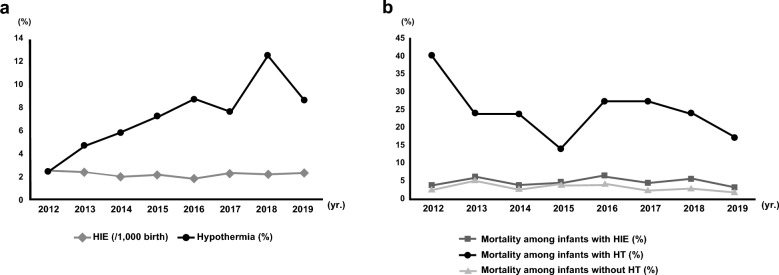
Incidence of hypoxic ischemic encephalopathy and the use of therapeutic hypothermia (**a**) and mortality (**b**) from 2012 to 2019. *HIE* hypoxic ischemic encephalopathy, *TH* therapeutic hypothermia.

According to the birth statistics, 3,188,372 live births occurred between 2012 and 2019. In total, 6994 infants were diagnosed with HIE. Of these, 470 infants were treated for TH during the study period. The HIE incidence in term infants was 2.4 per 1000 births (range:1.9 to 2.7), which did not change significantly during the study period. A 4.6% mortality was noted among the term infants with HIE, and this was maintained between 3.1 and 6.2% during the eight years included in this study. TH was performed on approximately 6.7% of infants with HIE, but the annual rate varied widely from 2.4 to 12.5%. Mortality among the infants with TH showed a significant reduction (Fig. 1).

Among the 6994 term infants diagnosed with HIE, inhaled nitric oxide therapy and invasive ventilation were applied to 61 infants (0.9%) and 486 infants (6.9%), respectively, and anti-epileptic drugs were administered to 287 infants (4.1%). CP was diagnosed in 526 infants (7.5%), delayed development in 823 infants (11.8%), autism spectrum disorders in 96 infants (1.4%), SNHL in 212 infants (3.0%), blindness in seven infants (0.1%), and seizures in 84 infants (1.2%). The combined outcome, which included CP, delayed development, SNHL, or blindness, affected 1122 infants (16.0%). Infants with HIE and TH showed significantly higher mortality than those with HIE but without TH, and the mortality was significantly higher at all ages after birth (p < 0.0001). Infants with HIE and TH also showed considerably higher morbidities, including DD, CP, SNHL, and seizures, and invasive treatment, including inhaled nitric oxide and ventilator care, compared to infants with HIE and without TH (p < 0.0001) (Table 1).

**Table 1:** Demographic characteristics and developmental outcomes among infants with hypoxic ischemic encephalopathy.

	Total (n = 6994)	HIE without TH (n = 6524)	HIE with TH (n = 470)	OR (95% CI)	*P* value
Mortality					
< 1 month	81 (1.2%)	54 (0.8%)	27 (5.7%)	7.30 (4.56/11.71)	< 0.0001
1–12 month	119 (1.7%)	84 (1.2%)	35 (7.4%)	6.17 (4.11/9.26)	< 0.0001
≥ 1 year	121 (1.7%)	73 (1.1%)	48 (10.2%)	10.05 (6.89/14.66)	< 0.0001
Total	321 (4.6%)	211 (3.2%)	110 (23.4%)	9.14 (7.09/11.78)	< 0.0001
iNO therapy	61 (0.9%)	39 (0.6%)	22 (4.7%)	8.17 (4.80/13.89)	< 0.0001
Invasive ventilator	486 (6.9%)	399 (6.1%)	87 (18.5%)	33.13 (22.80/48.13)	< 0.0001
Anti-epileptic drug					
Total	1377 (19.7%)	1077 (16.5%)	300 (63.8%)	8.93 (7.31/10.89)	< 0.0001
First prescription	287 (4.1%)	218 (3.3%)	69 (14.7%)	4.98 (3.73/6.65)	< 0.0001
Delayed development	823 (11.8%)	726 (11.1%)	97 (20.6%)	2.08 (1.64/2.63)	< 0.0001
Cerebral palsy	526 (7.5%)	453 (8.3%)	73 (15.5%)	2.47 (1.89/3.22)	< 0.0001
Autism spectrum disorders	96 (1.4%)	89 (1.4%)	7 (1.5%)	1.09 (0.50/2.37)	0.8218
SNHL	212 (3%)	186 (2.8%)	26 (5.5%)	2.03 (1.33/3.10)	0.0010
Blindness	7 (0.1%)	6 (0.1%)	1 (0.2%)	1.02 (0.80/1.32)	0.4816
ADHD	84 (1.2%)	80 (1.2%)	4 (0.9%)	0.69 (0.25/1.89)	0.4733
Seizure	1785 (25.5%)	1538 (23.6%)	247 (52.6%)	3.59 (2.97/4.34)	< 0.0001
CP or delayed development or SNHL or Blindness	1122 (16%)	981 (15%)	141 (30%)	2.42 (1.97/2.98)	< 0.0001
CP or delayed development or SNHL or blindness or mortality	1351 (19.3%)	1122 (17.2%)	229 (48.7%)	4.58 (4.78/5.54)	< 0.0001

*HIE* hypoxic ischemic encephalopathy, *TH* therapeutic hypothermia, *iNO* inhaled nitric oxide, *OR* odds ratio, *CI* confidence interval, *SNHL* sensorineural hearing loss, *ADHD* attention deficit hyperactivity disorder, *CP* cerebral palsy.

Trends of developmental outcomes among infants with hypoxic ischemic encephalopathy are in Table 2. The CP incidence decreased from 7.0 to 5.3% over the 8 years (P < 0.001). The incidence of autism spectrum disorder, ADHD, and seizure showed significant changes from 2012 to 2019. When dividing study periods into two groups, year 2012–2015 (4022 infants) and year 2016–2019 (2972 infants), a significant decrease in the number of infants with CP (7.9% vs 6.9%) was observed.

**Table 2:** Trends of developmental outcomes among infants with hypoxic ischemic encephalopathy.

	Total (n = 6994) (2012–2019)	*P* value*	2012–2015 (n = 4022)	2016–2019 (n = 2972)	*P* value**
Delayed development	823 (11.8%) (8.4–8.8%)	0.2244	459 (11.4%)	364 (12.2%)	< 0.001
Cerebral palsy	526 (7.5%) (7.0–5.3%)	0.0464	320 (7.9%)	206 (6.9%)	< 0.001
Autism spectrum disorder	96 (1.4%) (1.3–0.3%)	0.0043	70 (1.7%)	26 (0.9%)	0.426
SNHL	209 (3.0%) (3.9–2.9%)	0.0667	135 (3.4%)	74 (2.5%)	0.057
Blindness	1 (0.0%) (0.0–0.1%)	0.0924	0 (0.0%)	1 (0.0%)	
ADHD	84 (1.2%) (3.1–0.0%)	< 0.001	81 (2.0%)	3 (0.1%)	0.772
Seizure	1785 (25.5%) (19.2–19.0%)	< 0.001	948 (23.6%)	837 (28.2%)	< 0.001
Mortality	321 (4.6%) (3.4–3.1%)	0.179	179 (4.5%)	142 (4.8%)	0.002

*SNHL* sensorineural hearing loss, *ADHD* attention deficit hyperactivity disorder.

*The Cochran-Armitage trend test was used.

**χ^2^ test was used.

Based on the data from the disability identification system, brain lesion disorder was the most common disability in infants with HIE (5.7% in the HIE and without TH group vs. 14.7% in the HIE with TH group; p < 0.0001), followed by auditory disorder, intellectual disorder, and language disorder (Supplement Table 1).

**Figure 2:**
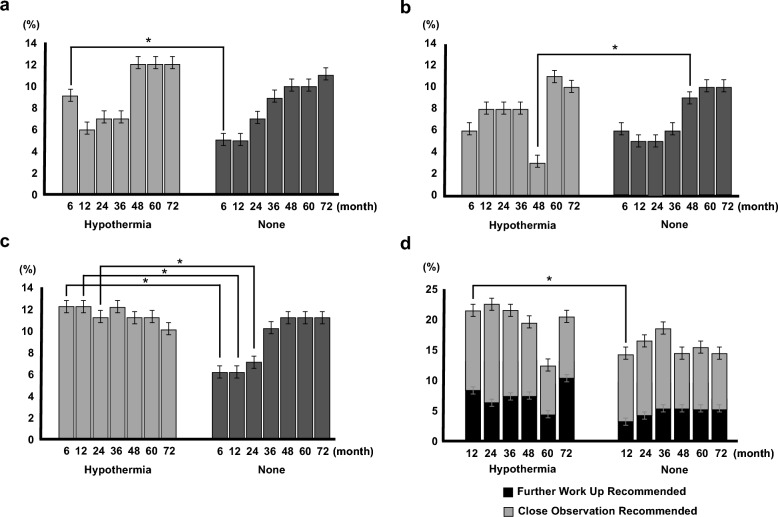
Comparisons of growth and developmental outcomes between Infants with and without therapeutic hypothermia. (**a**) Weight below10 percentile. (**b**) Height below 10 percentile. (**c**) Head circumference below 10 percentile. (**d**) The abnormal developmental screening outcome using the Korean Developmental Screening Test.

A total of 2045 infants (7.8%) with HIE showed a weight (WT) of < 10 percentile, 1777 infants (6.8%) showed a height (HT) of < 10 percentile and 2,263 infants (9.2%) showed a head circumference (HC) of < 10 percentile. There were significant variations in WT < 10 percentile (1.9–9.4%), HT < 10 percentile (3.2–8.8%), and HC < 10 percentile (6.2–9.8%) during the study period. Comparisons of poor growth outcomes between infants with and without TH were shown in Fig. 2a–c. The incidence of infants with WT < 10 percentile showed a significant difference only at 6 months, and the incidence of infants with HC < 10 was significantly different at 6 months, 12 month and 24 months of age. Although infants with TH demonstrated poorer conditions than those without TH, catch-up growth in HT, WT, and HC at 18 months was evident.

4.6% of infants with HIE required “further work-up,” and 10.6% required “close observation.” “Further work-up” (2.7–8.6%) and “close observation” (5.4–23.8%) varied significantly during the study period. Poor developmental outcomes between infants with HIE, with and without TH, are shown in Fig. 2d. Infants in the HIE with TH group required more “further work-up” (7.0% vs. 4.4%) and also more “close observation” (13.2% vs 10.9%). Exclusively, at the 12-month (p < 0.001), significantly poor developmental outcomes were demonstrated in the HIE with TH group.

The comparison of growth outcomes between infants with and without CP is shown in Table 3. Poor growth at the 2nd, 3rd, and 4th tests was significantly associated with an increased risk of CP regardless of TH.

**Table 3:** Comparisons of poor growth outcome at 12, 24, and 36 months of age between infants with and without cerebral palsy adjusted with therapeutic hypothermia.

	CP (n = 526)	No-CP (n = 6558)	OR (95% CI)	*P* value
HT below 10p at 12 month	38 (20.5%)	222 (4.8%)	5.03 (3.427–7.383)	< 0.0001
WT below 10p at 12 month	45 (24.3%)	210 (4.5%)	6.793 (4.713–9.793)	< 0.0001
HC below 10p at 12 month	69 (37.3%)	221 (26.5%)	11.368 (8.176–15.805)	< 0.0001
HT below 10p at 24 month	35 (22.6%)	226 (4.9%)	5.537 (3.707–8.272)	< 0.0001
WT below 10p at 24 month	47 (30.3%)	303 (6.6%)	6.274 (4.362–9.024)	< 0.0001
HC below 10p at 24 month	62 (40%)	296 (6.4%)	9.542 (6.768–13.454)	< 0.0001
HT below 10p at 36 month	42 (27.6%)	219 (5.5%)	6.512 (4.446–9.539)	< 0.0001
WT below 10p at 36 month	57 (37.5%)	301 (7.6%)	7.467 (5.261–10.599)	< 0.0001
HC below 10p at 36 month	71 (46.7%)	359 (9%)	8.835 (6.306–12.38)	< 0.0001

*CP* cerebral palsy, *OR* odds ratio, *CI* confidence interval, *HT* height, *WT* weight, *HC* head circumference, *p* percentile.

## Discussion

This population-based nationwide study showed that the HIE prevalence in term infants in Korea is similar to that in other developed countries. With the new introduction of TH as a treatment for HIE, poor neurodevelopmental outcomes and mortality showed decreasing trends; however, they remained in Korea. In addition, infants with HIE showed poor growth and developmental outcomes. Although catch-up growth was indicated as promising long-term outcomes, further efforts to improve outcomes for infants with HIE who treated TH or diagnosed CP should be needed.

The worldwide incidence of HIE varies between 1 and 3 per 1000 live births in developed countries and 2.3 to 30.6 per 1000 live births in developing countries^23–25^. The Korean incidence of 2.4/1000 live births is similar to the HIE incidence in the United Kingdom (2.63 in the 1990s, 2.96 per 1000 live births in the 2010s), which showed no significant changes over time^5,26^. Providing better access to medical and antenatal care in developing countries may reduce the incidence of HIE^23^. In contrast, in developed countries, significant improvement in the incidence is not usually observed once maternal care has settled down. Our results elucidated that the incidence of HIE in Korea has not changed over 8 years.

The mortality rate for newborns with HIE can vary depending on the severity of the condition and the availability of appropriate medical care. A study from Spain reported 21% mortality, which remained relatively constant during the study period from 2010 to 2019^27^. In Canada, 27% mortality in infants with HIE was noted during the study period (1988 to 2015)^28^. As a standard therapy of moderate to severe HIE, TH can reduce the mortality rate by 10–20% compared to control groups that did not utilize TH^29^. The United Kingdom study reported decreased mortality in infants with HIE who had undergone TH (12.9% to 6.7% from 2010 to 2017)^6^. In this study, it was impossible to compare the incidence due to the study's observational nature. However, we noted the mortality rate among infants with HIE and TH significantly declined from 40 to 16.9% during the eight years. In Korea, the overall mortality rate is low at 4.6%, which may be due to overestimation of HIE diagnosis due to retrospective ICD-10 code analysis.

TH was first introduced as standard therapy for moderate to severe HIE in Korea in the 2010s^9^. The use of TH in infants with HIE has remained under 10%, except for the year 2018. TH is broadly applied in up to 40.5% of all infants with HIE in the UK and 21.1% in the US^5,30^. The reasons for a significantly lower rate of TH in Korea are as follows: missed or underestimation of moderate to severe HIE as mild to no HIE, the ideal time point for the diagnosis of HIE passed, inability to transport the infant within the therapeutic window, or an active decision not to offer intensive care and lack of facilities or experienced TH specialist. Opportunities to explore practice-site variations and to develop quality improvement interventions to assure consistent, evidence-based care of term infants with HIE and the appropriate application of TH for eligible newborns should be considered^31^. It is promising that we should focus on building competence and designing quality improvement projects to increase the application of TH for newborns with HIE as an evidence-based practice.

HIE with TH group is affected by severe medical conditions compared to the HIE without TH group. Significantly higher mortalities and morbidities requiring invasive ventilators and anti-epileptic medication were found among HIE infants with TH than those without TH. The occurrence of persistent pulmonary hypertension in the newborn was between 13 and 25% in asphyxiated hypothermic infants^32,33^, which is higher than the incidence in the general population^34,35^. Also, neonates with hypothermia showed a 2.5 times higher risk of PPHN than controls^36^. In this study, 4.7% of infants with HIE and TH required inhaled nitric oxide treatment, which means it is 8.8 times more common than infants with HIE and without TH. This implies that either the baseline characteristics of the TH group were worse from the beginning or TH hurt the clinical course of PPHN itself.

It is encouraging to note that specific developmental outcomes, including CP, as a consequence of HIE, have decreased significantly in recent years in Korea. We assume that improvements in neonatal care and developmental follow-up protocols have led to better outcomes and the application of TH. Although other developmental products have not decreased significantly in the HIE with TH group, these results show that the active application of TH is promising for better outcomes.

TH significantly reduced the combined rate of death and severe disability in three trials that evaluated 18-month outcomes (risk ratio: 0.81, P = 0.002)^37^. Although there was a significant reduction in the rates compared to normothermia, hypothermia in survivors showed severe disability (28.1%), cerebral palsy (26.4%), deafness (4.7%), and mental and the psychomotor developmental index of less than 70 (26.5% and 26.2% respectively) with. We found that 21% of DD, 16% of CP, and 5.5% of SNHL were shown among the HIE infants treated with TH. Children with HIE scored significantly lower than typically developing children in fine motor skills, executive functions, memory, and language. In this study, infants in the HIE with TH group showed poor developmental outcomes (20.2%) in K-DST. Administering regular developmental screening and providing early intervention services should be needed. According to the National Health Screening Program for infants and children until 72 months of age, infants with HIE tended to have poor growth. Poor motor abilities, such as spasticity, can lead to difficulties in proper feeding, further exacerbating issues with development. This, in turn, can create a detrimental cycle that adversely affects neurodevelopment. This disparity is more evident in infants with HIE and TH, probably because of the seriousness of the HIE, than in infants without TH. An HC under the ten percentile was significantly higher in the HIE with TH group than in the HIE without TH group until the fourth test, corresponding to a chronological age of 30 months. This phenomenon may be attributed to infants with developmental problems being less likely to undergo general screening at the appropriate age. Alternatively, it is possible that the TH group missed the chance to experience growth during the rapid HC growth period before 36 months of age^38^.

The strength of this study is the use of data from nationwide databases, encompassing all live births and affected patients included in the study period. Long-term growth and developmental screening data until 6 years old were also analyzed.

The study has some limitations. The study contains weaknesses inherent to an observational study. National claim data did not include individual patient medical information. Specifically, the severity of HIE is indistinguishable. It is expected that the TH and HIE without TH groups would exhibit different distributions of Sarnat stages, although this information was inaccessible. In a real-world setting, mild HIE cases may be misclassified as moderate and cooled, especially in scenarios where timely decision-making within the therapeutic window is critical^39^. This adds a layer of complexity to the interpretation. Due to restricted access to individual data, nutrition status, and oral feeding difficulties, baseline growth parameters were not analyzed. It is impossible to establish the causality between CP or disabilities and poor growth in infants. The analyses relied on the accuracy of the included ICD codes, and labeling errors could not be identified and corrected. Due to the nature of ICD code search studies, we cannot differentiate the onset time of diseases. To prevent confusion, we excluded neonatal seizures from the seizure disorder category to focus on studying lifelong seizure disorders.

Nonetheless, understanding the timing of disease onset will offer valuable insights in the future. The KDST was used as a developmental screening tool, but the Bayley Scales of Infant Development was not used as a diagnostic tool. Moreover, the association of outborn birth status with mortality and morbidity was not evaluated.

With the new adaptation of TH treatment, neurodevelopmental outcomes showed decreasing trends; however, they remained in Korea. Further efforts and earlier interventions are warranted to improve infants' developmental and growth effects with HIE.

### Supplementary Information

Supplementary Table 1.

## Data Availability

The dataset analyzed in this study are not publicly available due to the policy of Research of Korea Centers for Disease Control and Prevention. However, dataset is available from the corresponding author on reasonable request.
