# Supplementary material for: Growth and developmental outcomes of infants with hypoxic ischemic encephalopathy
Source: Sci Rep. 2023 Dec 28;13:23100. doi: 10.1038/s41598-023-50187-0 (PMC10754824; doi:10.1038/s41598-023-50187-0)
Supplement: Supplementary file 1 — Supplementary Table 1. [file 41598_2023_50187_MOESM1_ESM.docx]

Supplement Table 1. Disabilities among infants with hypoxic ischemic encephalopathy

|  | HIE without TH(n=6524) | HIE with TH(n=470) | *P* value |
| --- | --- | --- | --- |
| Physical disorder | 11(0.2%) | 1(0.2%) | 0.8985 |
| Brain lesion disorder | 374(5.7%) | 69(14.7%) | <.0001 |
| Visual disorder | 6(0.1%) | 1(0.2%) | 0.4816 |
| Hearing disorder | 29(0.4%) | 9(1.9%) | 0.0001 |
| Speech disorder | 51(0.8%) | 4(0.9%) | 0.9829 |
| Intellectual disorder | 82((1.3%) | 6(1.3%) | 0.8481 |
| Autistic disorder | 21(0.3%) | 2(0.4%) | 0.8041 |
| Kidney disorder | 2(0.03%) | 2(0.4%) | 0.0011 |
| Liver disorder | 1(0.02%) | 0 | 0.7782 |
| Facial disorder | 0 | 1(0.2%) | 0.0004 |
| Epileptic disorder | 3(0.05%) | 0 | 0.6265 |

Abbreviations: HIE hypoxic ischemic encephalopathy; TH therapeutic hypothermia
